# Electric and Dielectric Properties in Low-Frequency Fields of Composites Consisting of Silicone Rubber and Al Particles for Flexible Electronic Devices

**DOI:** 10.3390/ma15062309

**Published:** 2022-03-21

**Authors:** Alexandrina Teusdea, Iosif Malaescu, Paula Sfirloaga, Catalin Nicolae Marin

**Affiliations:** 1 Faculty of Physics, West University of Timisoara, Bd. V. Parvan 4, 300223 Timisoara, Romania; alexandrina.teusdea68@e-uvt.ro (A.T.); iosif.malaescu@e-uvt.ro (I.M.); 2 National Institute for Research and Development in Electrochemistry and Condensed Matter, Str. P. Andronescu 1, 300224 Timisoara, Romania; paulasfirloaga@gmail.com

**Keywords:** composite, silicon rubber, aluminum, electrical conductivity, complex dielectric permittivity, electrical modulus, flexible electronics

## Abstract

Understanding the electrical conduction and dielectric polarization properties of elastomer-based composites is important for the design of flexible and elastic electronic devices and circuits. Five samples were manufactured by mixing silicone rubber (RTV-530) with Al particles in different volume fractions, x equal to 0%, 0.5%, 1%, 2.5% and 5.1%. Using the complex impedance measurements, the electric modulus, M, the electrical conductivity, σ, and the dielectric permittivity, ε, over the frequency range 100 Hz–200 kHz were analyzed. The electrical conductivity spectrum, σ(f), follows the Jonscher universal law and the DC conductivity of the samples, σ_DC_, increases from 2.637·10^−8^ S/m to 5.725·10^−8^ S/m, with increasing x from, 0 to 5.1%. The conduction process was analyzed in terms of Mott’s variable-range-hopping (VRH) model. The hopping distance of the charge carriers, R_h_ decreases with increasing x, from 7.30 nm (for x = 0) to 5.92 nm (for x = 5.1%). The frequency dependence of permittivity, ε(f) = ε′(f) − iε″(f), reveals a relaxation process with the maximum of ε″(f) shifting from 301 Hz to 385 Hz and values of ε′(f) increasing with the increase of x.

## 1. Introduction

In recent years, there has been an increasing interest in composite materials, such as nanofluids, gels or elastomers, which have been intensively studied for practical applications [1,2]. A composite material can be produced by dispersing nano-/microparticles in a liquid or solid matrix [3,4] and thus can provide improved performance compared to its components [3]. Recently, a special interest has been observed for composite materials consisting of metal oxide particles dispersed in a polymer matrix [5]. The electrical and dielectric properties of this type of composite material depend on the properties of the matrix and on the nature, size and concentration of the dispersed particles [6]. In Ref. [7], the authors show that by combining the graphite nanoplatelets and magnetite with different types of polymer, new composite materials can be developed with very good microwave properties. Moreover, other composites such as those obtained by mixing of BaTiO_3_ particles with a thermoplastic material [8] or using a thermoresistant polymer [9] as a matrix were found to be useful in applications with miniaturized electronic devices and sensors [10]. By mixing metal particles such as Fe or Ni with natural rubber, elastomers with improved electrical and magnetic properties are obtained [11]. Other elastomers obtained by introducing magnetite (Fe_3_O_4_) or NiZn ferrite particles in acrylonitrile butadiene rubber provide good protection against electromagnetic interference in the frequency range 1–12 GHz [12]. 

Silicone rubber (SR) is a dielectric elastomer [13] often constituting a matrix for obtaining composite materials. SR has low stiffness and high breakdown strength and generally can be used in sealing applications [14]. In order to be used in different applications, the silicone rubber is mixed with micro- or nanoparticles of different types (ceramic particles, conductive particles or highly polarizable polymers [15]). This contributes to the change of dielectric constant of the composite based on silicon rubber and makes it useful in applications such as elastic and flexible charge-storage devices or actuators [16]. The existing studies mostly focus on the thermal conductivity [17,18,19] or dielectric properties [17] of Al particle-reinforced silicone rubber composites [17,18], with Al_2_O_3_ particles [19] or with TiO_2_ particles [20]. These studies show that such composites can have potential applications in electromechanical actuators because of their high values of thermal conductivity or dielectric permittivity and their good elasticity. The determination of the electrical conductivity (σ) or dielectric permittivity (ε) of composites made of silicone rubber and Al particles, and experimental studies regarding the dependence both of (σ) and (ε) on the volume fraction of Al particles dispersed in silicone rubber are few in the literature. In this context, experimental measurements of the electrical conductivity and dielectric permittivity for composites consisting of silicone rubber and Al particles are very important and useful, in order to know whether the studied materials could be used in various applications, such as flexible electric and electronic devices.

In this paper we report the manufacture of some composite samples obtained by mixing silicone rubber with Al microparticles in different concentrations. The measurements of complex impedance (Z) over the frequency range 100 Hz–0.2 MHz allowed the determination of the electrical conductivity (σ) of composite samples and of their electrical modulus (M) and complex dielectric permittivity (ε = ε′ − iε″). The electrical conductivity was analyzed via Nyquist plots and in terms of Mott’s variable-range-hopping (VRH) theoretical model and the dielectric permittivity exhibits a low-frequency relaxation process. The results can be useful in the design of flexible and elastic electronic device and circuits.

## 2. Materials and Methods

For this study, we prepared a composite material using a commercial RTV-530 silicone rubber (SR) from Prochima [21] with the density $\rho_{SR}=1.30\;g/cm^{3}$ and aluminum powder from Sigma Aldrich [22] with particles of micrometer sizes (smaller than 25 μm) and density $\rho_{AL}=2.7\;g/cm^{3}$. The silicone rubber (SR) is a nontoxic elastomer and is prepared with two components (A and B). These components are mixed in equal quantities and the result obtained after a polyaddition reaction is a rubber with medium elasticity and hardness. 

**Figure 1:**
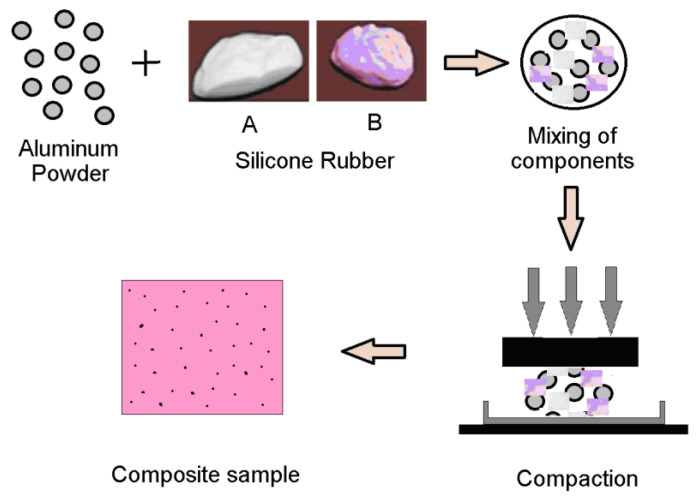
The stages of preparation process of the composite samples of silicone rubber with Al particles.

For the preparation of the composite samples, we mixed a quantity M_xAl_ of aluminum particles with a quantity M_SR_ of silicone rubber (equal quantities M_SR_/2 of each component A and B). The obtained mixture was placed in a parallelepiped mold and pressed for 2–3 min until it took the shape of the mold, and after 24 h the composite sample was obtained. Figure 1 shows the stages of the obtaining process of samples.

The following quantities of aluminum particles, M_xAl_ and of silicone rubber, M_SR_, were used to obtain the samples (SR-xAl):

- for sample A: and (10 g of each component, A and B);
- for sample B: and (9.9 g of each component, A and B);
- for sample C: and (9.8 g of each component, A and B);
- for sample D: and (9.5 g of each component, A and B);
- for sample E: and (9 g of each component, A and B).

**Figure 2:**
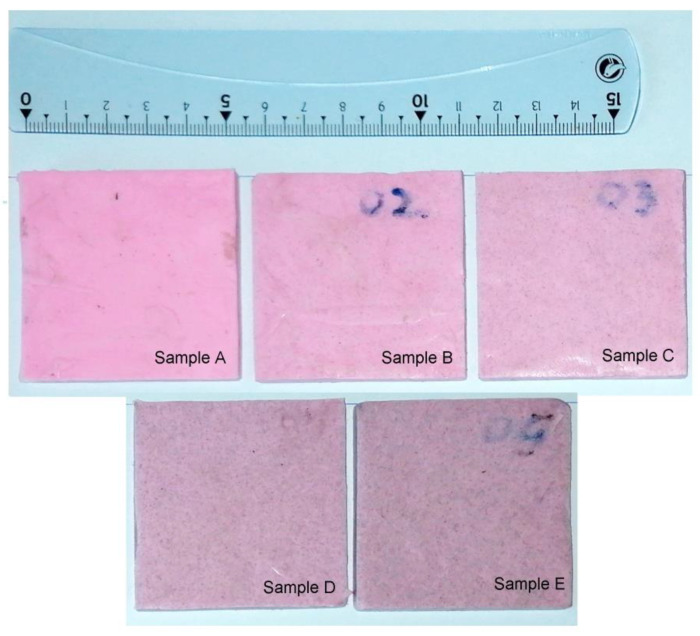
The images of the SR—xAl composite samples of silicone rubber with Al particles (x—volume fraction of Al particles): sample A (x = 0); sample B (x = 0.005); sample C (x = 0.01); sample D (x = 0.025); and sample E (x = 0.051).

The obtained composite samples have the shape of a square parallelepiped with a side of 5.5 cm and a thickness of 0.5 cm (Figure 2). Considering the quantities of materials used in the preparation of samples and their densities (ρ_Al_ and ρ_SR_), we can determine the total volume of the composite in each case, V_tot_ = V_X,Al_ + V_SR_, so that the volume fraction of Al particles dispersed in the silicone rubber is x = V_X,Al_/V_tot_. The x values obtained for each composite sample are: x(A) = 0; x(B) = 0.5%; x(C) = 1%; x(D) = 2.5%; x(E) = 5.1%. 

Morphological analysis was performed using the INSPECT S scanning electron microscope (SEM, FEI Europe B.V., Eindhoven, The Netherlands), equipped with an energy-dispersive X-ray detector (EDX).

**Figure 3:**
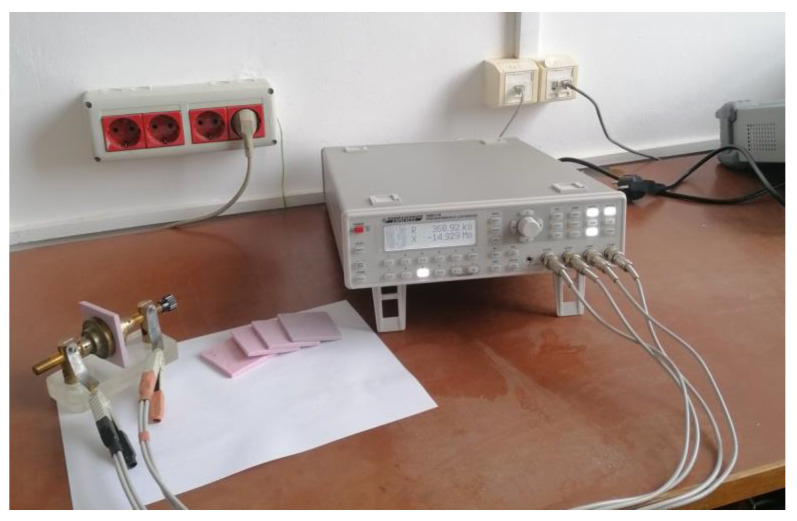
The photo with the used experimental setup for measurements.

The samples were introduced in a planar capacitor with circular plates having the diameter of 4 cm and a variable distance d, between the plates, connected to an RLC-meter [23,24]. Figure 3 shows the photograph with the measuring installation. At the room temperature, the real component Z′ and imaginary component Z″ of the complex impedance of each sample were measured. Based on the impedance measurements, the electrical modulus, the electrical conductivity and the complex dielectric permittivity of the samples were determined. 

## 3. Results and Discussion

### 3.1. SEM–EDX Results

**Figure 4:**
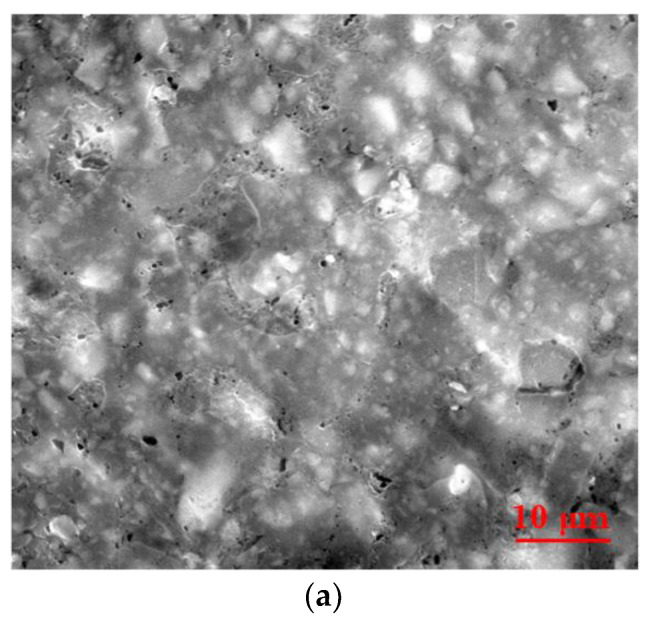

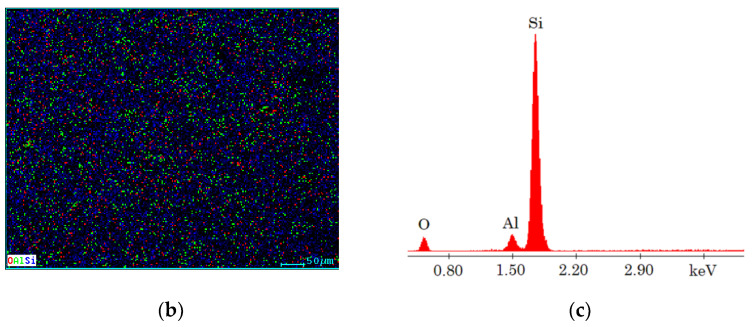
(**a**) SEM image of composite sample D; (**b**) element mapping; and (**c**) EDX spectrum for sample D.

The SEM image of sample D, element mapping and EDX spectrum of this sample are presented in Figure 4a–c. From the SEM image (Figure 4a), the presence of Al particles and their agglomerations inside the silicone rubber is observed. Moreover, to evaluate the elemental composition of the composite sample, a semiquantitative analysis was performed by EDX and from this analysis of the EDX spectrum (Figure 4c) it can be observed that the analyzed sample contains the following elements: O, Al and Si. At the same time, from the mapping, uniform distribution of elements in the studied material can be seen (Figure 4b). The SEM investigation of the other samples (B, C and E) leads to similar results (the samples contain O, Al and Si, the Al particles are well distributed in the samples and their agglomerations inside the silicone rubber are observed).

### 3.2. Complex Impedance and Electrical Modulus

**Figure 5:**
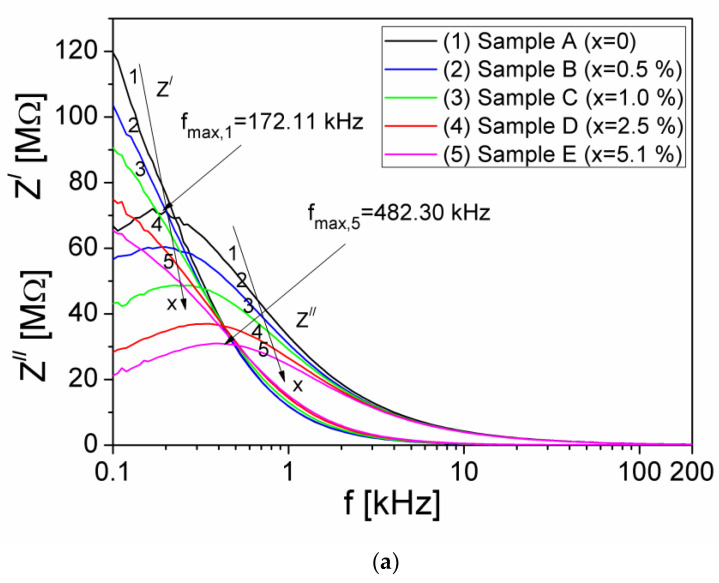

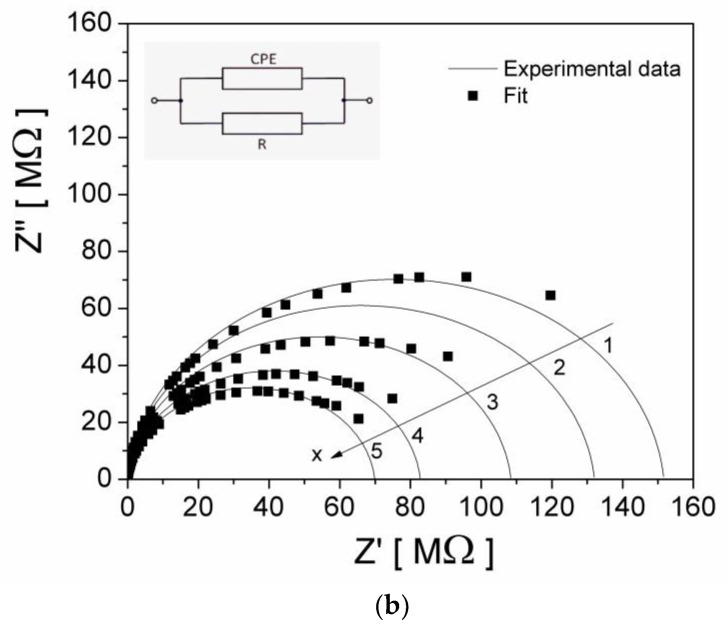
(**a**) The frequency dependence of the real (Z′) and imaginary (Z″) components of the complex impedance of the investigated composite samples at different volume fractions x of Al particles; (**b**) the Nyquist plots of the impedance spectra of samples and their according fitting with the electrical equivalent circuit.

Figure 5a shows the frequency dependence of the real (Z′) and imaginary (Z″) components, of the complex impedance of the composite samples, over the frequency range (100 Hz–200 kHz) and at different volume fractions x, of Al particles dispersed in the silicone rubber and Figure 5b shows the Nyquist representations, Z″(Z′) for each sample in the frequency range investigated.

As can be seen in Figure 5a, at low frequencies up to 1 kHz, the real component Z′ decreases with increasing of the x volume fraction of Al particles from the composite sample. Moreover, at a constant volume fraction x of Al particles and frequencies up to 1 kHz, the real component Z′ decreases rapidly with increasing the frequency, after which it decreases very little, by increasing the frequency up to 200 kHz. The rapid decrease of the Z′ component at frequencies up to 1 kHz for all composite samples (see Figure 5a), may be due to the polarization processes [25] that take place in the composites. This leads to a corresponding increase in the electrical conductivity of the composite sample by increasing the x volume fraction of the Al particles in the composite material.

By increasing the frequency, the charge carriers from samples will no longer be able to follow the rapid oscillations of the electrical field, so that their oscillation will remain behind the electrical field, which determines a slower decrease of the Z′ component [4] of the complex impedance, as can be seen in Figure 5a.

The imaginary component Z″ presents a maximum at a frequency *f_max,(Z)_* which moves towards higher values when the volume fraction, x increases. The appearance of this maximum shows the existence of an electrical relaxation process [5,6] due to the presence of charge carriers in the investigated samples. Using the Debye equation, $2\pi \;f_{\max,(Z)}\tau_{Z}=1$ [26] and the experimental values of the $f_{\max,(Z)}$, from Figure 5a we have computed the relaxation times $\tau_{Z}$, corresponding to each x volume fraction of Al particles from the samples. The obtained values are listed in Table 1.

From Figure 5b, the impedance spectrum or the Nyquist plots are characterized by the appearance of semicircular arcs for all the samples. The diameter of semicircle decreases with the increase the volume fractions x of Al particles, which determines an increase of the σ_DC_ electrical conductivity. The existence of a single semicircle for all volume fractions x of Al particles shows that the electrical process in the samples is characterized by a single relaxation mechanism, being correlated with the microstructure of the sample when it is modeled in terms of equivalent electrical circuit [27,28]. The experimental data of the Nyquist plots, Z″(Z′) from Figure 5b, for the investigated samples were fitted with a theoretical dependence, Z″(Z′), and the best fit of these plots was given by an electrical model, presented in the inset of Figure 5b. This electrical model, corresponding to each sample, consists of a parallel group of R-CPE (R-CPE stands for Resistor—Constant Phase Element), being in accordance with the microstructure of samples. The parameters of the fit for each sample are shown in Table 1, where *R* represents the parallel resistance, whilst *P* and *k* are parameters of the impedance of CPE, corresponding to each sample, given by the equation: $Z_{CPE}=P^{-1}{\left(i\cdot 2\pi f\right)}^{-k}$, where $i=\sqrt{-1}$.

From the Table 1, it is observed a decrease of the relaxation time τ_Z_ by increasing the volume fraction of Al particles in the samples, from 0% to 5.10%.

To investigate the relaxation mechanism of electrical conduction in the samples, another important parameter that can be used is the electrical modulus, *M*, whose real (*M′*) and imaginary (*M″*) components can be determined based on the experimental measured values *Z′* and *Z″* of the complex impedance (see Figure 4) with the relations [27]:

(1) $$M^{\prime }=\left(\frac{\omega \varepsilon_{0}A}{d}\right)\cdot Z^{\prime \prime }$$

(2) $$M^{\prime \prime }=\left(\frac{\omega \varepsilon_{0}A}{d}\right)\cdot Z^{\prime }$$

Here, *d* and *A* are the dimensions of the sample in the capacitor (thickness *d* = 5 mm and the transversal sectional area, *A* = 12.56 cm^2^), *ω* is the angular frequency of the electric field and *ε*_0_ is the free space permittivity. The analysis based on the electrical modulus provides a better separation between dielectric relaxation and electrical conduction losses than the analysis based solely on dielectric permittivity [29,30].

**Figure 6:**
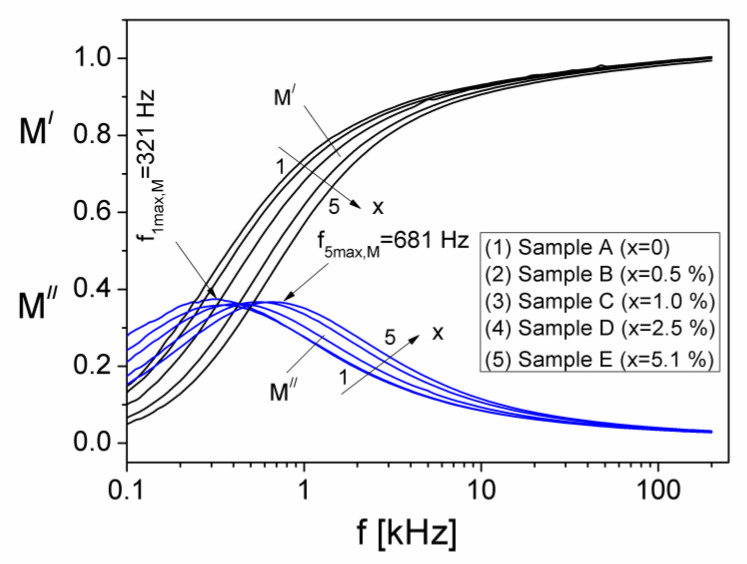
The frequency dependence of the real (M′) and imaginary (M″) components of the electric modulus of the samples with different volume fractions, x of Al particles.

**Figure 7:**
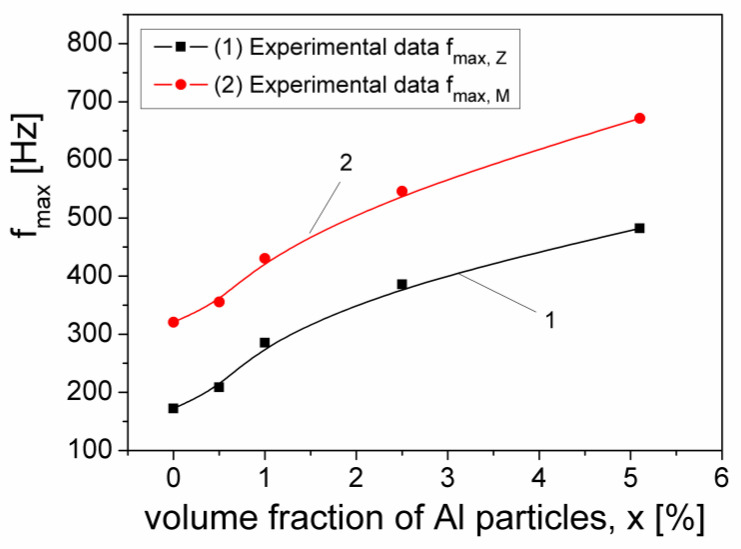
The volume fraction dependence of the f_max,(Z)_ and f_max,(M)_ frequencies, corresponding to the imaginary components Z″ and M″, respectively.

In Figure 6 we have shown the frequency dependence of the components M′ and M″, at different volume fractions, x of Al particles, from the samples. From Figure 6 it can be observed that at low frequency, M′ is small for all x volume fractions of the Al particles dispersed in the silicone rubber, which shows that the polarization effects at interface electrode/sample are reduced [30,31]. At each volume fraction x, M′ increases continually and tends to a constant value of 0.98 by increasing the frequency of the electrical field. At the same time, by increasing the frequency, the magnitude of M′ decreases slightly with increasing x, which may be attributed to an Al concentration dependent relaxation process in the composite samples. The imaginary component M″ presents a maximum for each volume fraction, x, corresponding to a relaxation frequency, f_max,(M)_ which moves from 321 Hz to 681 Hz when x increases from x = 0 to x = 5.1%, which shows a shift towards frequencies greater than the frequencies corresponding of the maximum f_max,(Z)_ of the imaginary component Z″, of the complex impedance [32], as can be seen in Figure 7.

Using the experimental values of the f_max,(M)_ and Debye equation, we have computed the relaxation times, $\tau_{M}$ corresponding to each volume fraction x, of Al particles dispersed in the samples. The obtained values are listed in Table 1.

### 3.3. The Electrical Conductivity

Using the experimental values Z′ and Z″ of the complex impedance (Figure 5), the electrical conductivity σ, of the composite samples was computed with the relation (3), where, $\left|Z\right|=\sqrt{{Z^{\prime }}^{2}+{Z^{\prime \prime }}^{2}}$, is the complex impedance modulus of the sample.

(3) $$\sigma =\frac{Z^{\prime }}{{\left|Z\right|}^{2}}\cdot \frac{d}{A}$$

**Figure 8:**
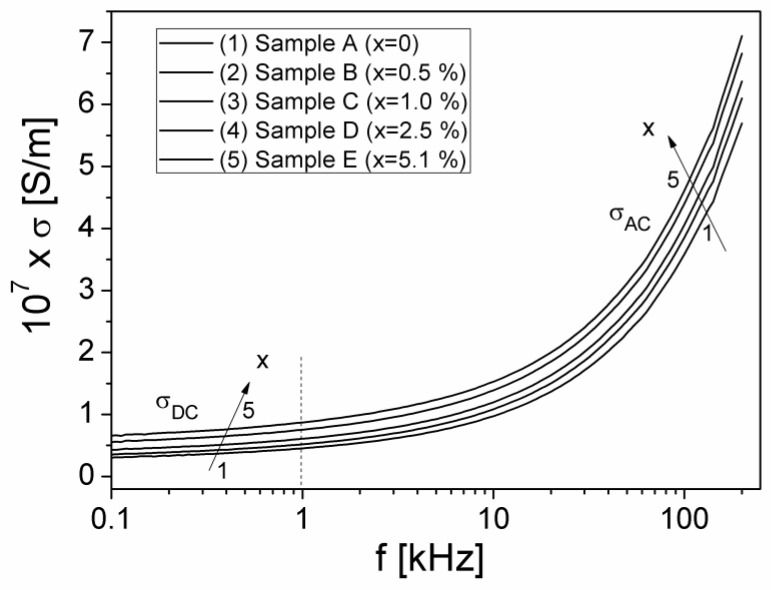
The frequency dependence of the conductivity σ, at different volume fractions, x of the Al particles dispersed in the composite material.

The frequency dependence of σ, in the frequency range 100 Hz to 200 kHz and at different volume fractions x of the Al particles dispersed in the composite material, is shown in Figure 8.

As can be seen from Figure 8, at a constant volume fraction x, the conductivity spectrum, σ(f) consists of two regions: (1) a region in which σ tends to a constant value by decreasing the frequency (at very low frequencies, σ does not depend on frequency) corresponding to DC-conductivity (σ_DC_); (2) a dispersion region, where σ rapidly increases with frequency, corresponding to AC-conductivity (σ_AC_). A similar frequency dependence σ(f) was also observed for other composite samples, such as Cu_1+x_Mn_1-x_O_2_ type composite materials (with x = 0–0.10) [33], or polymers of PTB7 type [34]. This behavior is in accordance with the Jonscher universal law [35]:

(4) $$\sigma (\omega )=\sigma_{DC}+\sigma_{AC}$$

As seen in Figure 8, in the first region of the low frequency range, the measured conductivity decreases slowly by the decrease of frequency, tending to a constant value, which will not depend on the frequency, representing the DC component of conductivity (σ_DC_) according with the Jonscher universal law [35]. The numerical values of the DC conductivity were determined based on the Nyquist impedance diagrams, Z″(Z′) in Figure 5b) and the fit parameters (see Table 1) corresponding to the equivalent electrical model for each sample [34,36]. As a result, the values thus obtained for σ_DC_ are listed in Table 1. It is observed that the variation of volume fraction from x = 0 to x = 5.1%, leads to an increase in the conductivity of the samples from 2.637·10^−8^ S/m to 5.725·10^−8^ S/m. The values obtained for the DC-conductivity were compared with the values obtained by other authors in the paper [17], for reinforced silicone rubber composites sampled with Al particles, being of the same order of magnitude.

The AC-conductivity component ($\sigma_{AC}$) depends on frequency, being correlated with the relaxation processes determined by the localized electric charge carriers from sample [35], and is given by the relation:

(5) $$\sigma_{AC}=A_{0}\omega^{n}$$

Here, *n* is an exponent, which is dependent on both frequency and temperature (0 < *n* < 1) and *A*_0_ is a pre-exponential factor [37]. Taking into account Equations (4) and (5), another form of Jonscher’s universal law can be written:

(6) $$\sigma (\omega )=\sigma_{DC}+A_{0}\omega^{n}=\sigma_{DC}\left[1+{\left(\frac{\omega }{\omega_{c}}\right)}^{n}\right]$$

Here, $\omega_{c}$ represents the crossover frequency and the pre-exponential factor is $A_{0}=\omega_{c}^{-n}\sigma_{DC}$ [35,37]. On the other hand, $\omega_{c}$ represents the frequency of transition from the DC regime to the dispersive regime (from high frequencies), according to Jonscher’s universal law [35].

**Figure 9:**
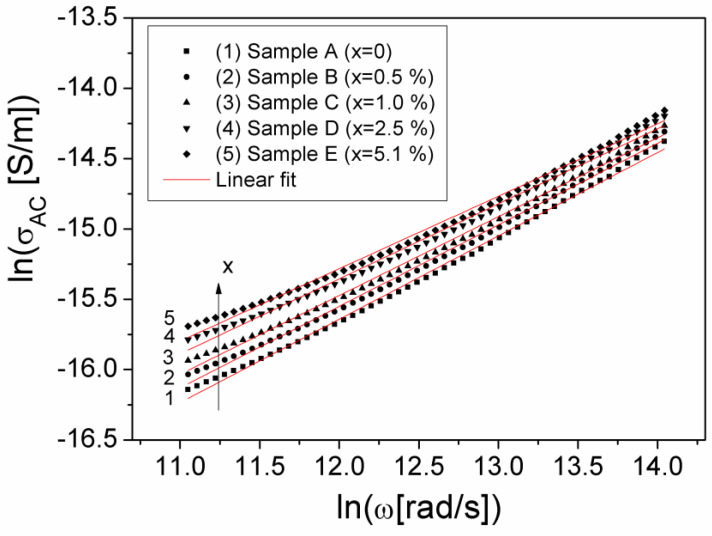
The lnσ_AC_(lnω) dependence at different volume fractions x of Al particles of the samples.

By the logarithm of Equation (5), a linear dependence between lnσ_AC_ and lnω results, which is presented in Figure 9 for the investigated samples.

The experimental dependence ln(σ_AC_)(ln(ω)), from Figure 9, was fitted with a straight line, thus being able to determine both the parameter A_0_ and the exponent n, corresponding to each sample, the obtained values being showed in Table 1. From Table 1, it is observed that the exponent n decreases slightly, from 0.593 for sample A (x = 0) to 0.515 for sample E (x = 5.1%). This low variation of n indicates a constant dispersion of σ_AC_ conductivity with frequency by increasing the volume fraction of Al particles from samples (see Figure 8). Taking into account the values obtained for A_0_ and n, as well as the conductivity σ_DC_, we determined the crossover frequency, ω_c_ corresponding to each volume fraction of Al particles in the composite samples, the values obtained being presented in Table 1.

It is known that in the electrical conduction mechanism by hopping of charge carriers [38,39] and based on the Mott’s variable-range-hopping (VRH) model [40], the relationship between the conductivity *σ_DC_* and the hopping frequency, *ω_c_*, is given by:

(7) $$\sigma_{DC}=\left(\frac{N_{C}\cdot e^{2}\cdot R_{h}^{2}}{12\pi \cdot k_{B}T}\right)\cdot \omega_{c}$$

Here, *e* is the electric charge of the electron; *N_C_* is the effective concentration of charge carriers [40,41] from the sample; *k_B_* is the Boltzmann constant: *T* is the absolute temperature and *R_h_* is the hopping distance [39]. From the VRH model, the hopping distance is given by:

(8) $$R_{h}={\left(\frac{9}{8\alpha k_{B}T\cdot N(E_{F})}\right)}^{1/4}$$

In Equation (8), $\alpha ≅10^{7}\;cm^{-1}$ represents the degree of localization [39] and *N(E_F_)* is the density of the localized states at the Fermi level E_F_ [40,41]. Taking into account that N_C_ and *N*(*E_F_*)** are correlated by the relation, $N_{C}=k_{B}T\cdot N(E_{F})$ [41,42] and using Equations (7) and (8) we established the following equation that allows the determination of the hopping distance based on electrical conductivity measurements:

(9) $$R_{h}=\frac{3}{4}{\left(\frac{e^{2}\omega_{c}}{6\pi \alpha k_{B}T\cdot \sigma_{DC})}\right)}^{1/2}$$

Considering the obtained values for $\omega_{c}$ and $\sigma_{DC}$ (see Table 1) and room temperature T = 300 K, with Equation (9) we have computed the hopping distance R_h_ corresponding to each sample, and the obtained values are shown in Table 1. From Table 1, it is observed that the hopping distance, *R_h_*, decreases with the increase in Al particles’ volume fraction in the samples, from 7.30 nm (for x = 0) to 5.92 nm (for x = 5.1%). Moreover, one can observe that for all samples, the hopping distance R_h_ is smaller than the Al particles’ size (that are in the order of micrometers), which means that the conduction mechanism in low frequencies is realized by the hopping process between the localized states within the silicon rubber or between the localized states at the Al particles’ surfaces.

### 3.4. The Dielectric Permittivity

As is known, the electrical modulus, *M*, can be defined by Equation (10), where $\varepsilon^{\prime }$ and $\varepsilon^{\prime \prime }$ represent the real and imaginary components, respectively, of the complex dielectric permittivity of the sample, $\varepsilon =\varepsilon^{\prime }-i\varepsilon^{\prime \prime }$ [43].

(10) $$\underline{M}=\frac{1}{\varepsilon^{\prime }-i\varepsilon^{\prime \prime }}=M^{\prime }-iM^{\prime \prime }$$

From Equation (10), the $\varepsilon^{\prime }$ and $\varepsilon^{\prime \prime }$ components of the complex dielectric permittivity can be determined. The following relationships result:

(11) $$\varepsilon^{\prime }=\frac{M^{\prime }}{{M^{\prime }}^{2}+{M^{\prime \prime }}^{2}}$$

(12) $$\varepsilon^{\prime \prime }=\frac{M^{\prime \prime }}{{M^{\prime }}^{2}+{M^{\prime \prime }}^{2}}$$

**Figure 10:**
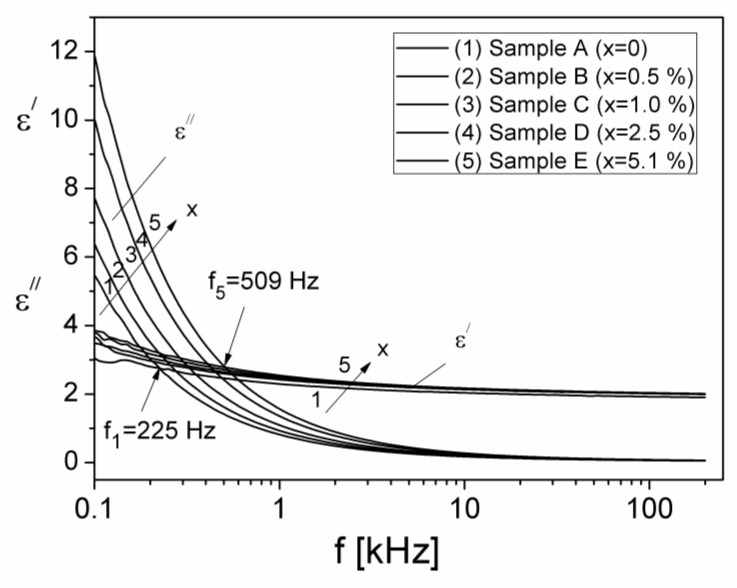
The frequency dependence of the real, ε′, and imaginary, ε″, components of the complex dielectric permittivity of the composite samples containing different volume fractions, x, of the Al particles.

Knowing the values $M^{\prime }$ and $M^{\prime \prime }$ of the electric modulus M (Figure 6), obtained from the complex impedance measurements and using Equations (11) and (12), the frequency dependencies of $\varepsilon^{\prime }$ and $\varepsilon^{\prime \prime }$ components were determined and plotted in Figure 10.

From Figure 10 it is observed that the real component, ε′, increases from 3.0 to about 3.8, at 100 Hz, by increasing the volume fraction of Al particles, whilst by increasing the frequency, ε′ decreases to the approximate value $\varepsilon^{\prime }≅2$, for all samples. For a constant volume fraction of Al particles, x, the imaginary component, ε″, decreases sharply with increasing frequency. Moreover, at the frequency of 100 Hz, ε″ increases from 5.5 to about 11.8, by increasing the volume fraction x. At the same time, from Figure 10 it is observed that at low frequencies, the values of the ε″ component are much higher than those of the real component ε′, thus indicating high conduction losses in the composite samples or electrode polarization process. At frequencies larger than 500 Hz, ε″ is smaller than ε′ for all samples, indicating that the dielectric relaxation becomes prevalent.

The total dielectric losses in a material [43] are due both to electrical conduction (${\varepsilon^{\prime \prime }}_{c}$) and dielectric relaxation (${\varepsilon^{\prime \prime }}_{rel}$):

(13) $$\varepsilon^{\prime \prime }(\omega )={\varepsilon^{\prime \prime }}_{c}(\omega )+{\varepsilon^{\prime \prime }}_{rel}(\omega )$$

**Figure 11:**
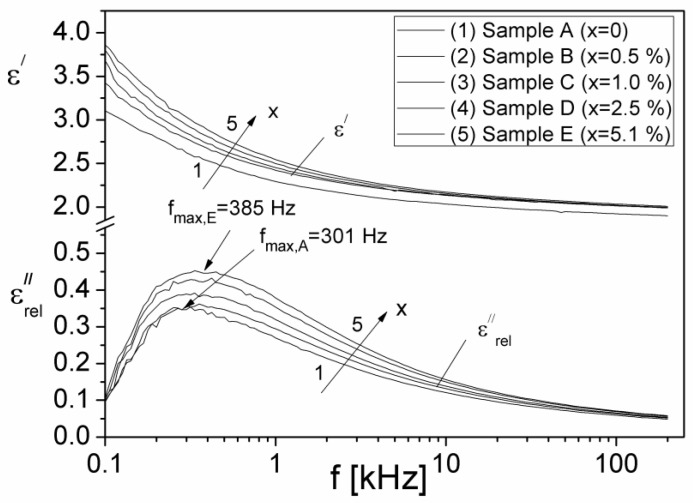
Frequency dependence of the real component ε′ and of the imaginary component ${\varepsilon^{\prime \prime }}_{rel}$ due to the dielectric relaxation of samples.

The component, ${\varepsilon^{\prime \prime }}_{c}$, due to conduction [43] can be expressed by the relation ${\varepsilon^{\prime \prime }}_{c}=\sigma /2\pi f\varepsilon_{0}$, where σ is the electrical conductivity of the investigated sample. If we assume that the value of σ is equal to the value of $\sigma_{DC}$ for each composite sample (see Table 1), we can determine the component ${\varepsilon^{\prime \prime }}_{c}$, and then from Equation (13) the component ${\varepsilon^{\prime \prime }}_{rel}$ corresponding to the dielectric relaxation can also be computed. In Figure 11, the frequency dependencies of the components ε′ and ${\varepsilon^{\prime \prime }}_{rel}$ are shown.

From Figure 11, it is observed that the imaginary component ${\varepsilon^{\prime \prime }}_{rel}$, corresponding to the dielectric relaxation processes in the samples, presents a maximum for each volume fraction x at the relaxation frequency, f_max_, which moves from 301 Hz to 385 Hz, when x increases from x = 0 to x = 5.1%. Using the Debye equation and the experimental values of f_max_ (Figure 11) we have computed the relaxation times due to the dielectric relaxation processes, τ_rel_, for each investigated sample. The obtained values are listed in Table 1. The values of τ_rel_ are close to those of τ_M_, thus confirming the fact that the analysis of the frequency dependence of the electrical module, *M(f) = M′(f) − iM″(f)*, is useful in the study of dielectric relaxation processes in the case of conductive samples, for which ${\varepsilon^{\prime \prime }}_{c}=\sigma /2\pi f\varepsilon_{0}$ has high value that covers the dielectric relaxation maximum.

**Figure 12:**
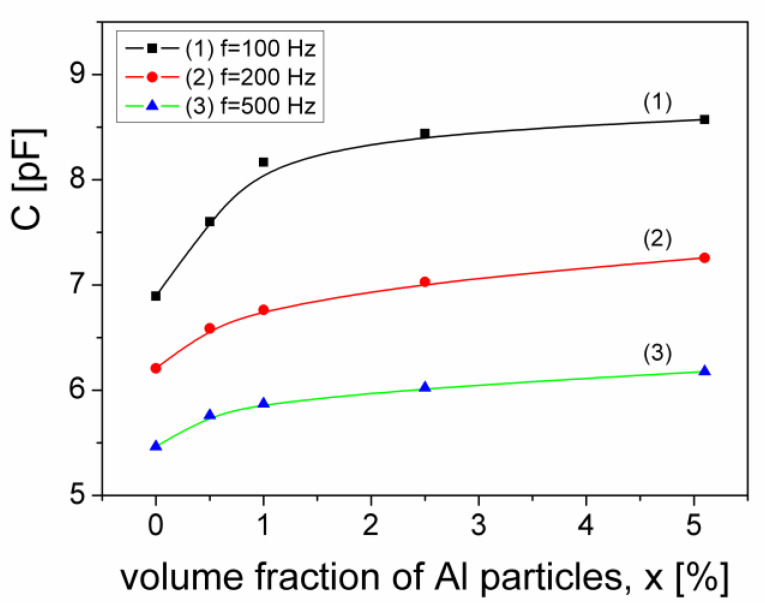
The volume fraction dependence of the electrical capacitance of a capacitor with the investigated composite samples as dielectric, at three frequencies.

Considering a planar capacitor that has between its armatures the composite samples investigated in this paper, we examined the capacitance $C={\varepsilon^{\prime }}_{sample}C_{0}$, as a function of volume fraction, x of Al particles dispersed in the samples, for several frequencies from the low frequency range (0.1–1) kHz. We denoted with C_0_ the capacity of the planar capacitor in the absence of the sample between the armatures, whose value is C_0_ = 2.22 pF. In Figure 12 it is presented the variation of the capacitance, C, depending on the volume fraction of Al particles dispersed in the composite sample, for three frequencies from the low frequency range: 100 Hz, 200 Hz and 500 Hz.

From Figure 12, it results that for all three frequencies, the capacitance, C increases by increasing the volume fraction of Al particles in the composite sample, from a minimum value C_x0_ (corresponding to the volume fraction, x = 0) to a maximum value C_xmax_ (corresponding to the volume fraction, x = 5.1%). The maximum relative change of the electrical capacitance, (C_xmax_ − C_x0_)/C_x0_, decreases by increasing the frequency from 24% (at 100 Hz) to 13% (500 Hz), as can be seen in Table 2.

Therefore, this result shows that the capacitance of a capacitor filled with composite material can be tuned by the volume fraction of aluminum particles dispersed in the composite, which is useful in flexible electronics applications.

## 4. Conclusions

Based on the complex impedance measurements, over the frequency range (100 Hz–200 kHz), the electrical conductivity (σ) and the dielectric permittivity (ε) of composite samples consisting of silicone rubber and aluminum particles, in different volume fractions x = (0%; 0.5%; 1%; 2.5% and 5.1%), were investigated.

The values of the DC-conductivity (σ_DC_) were determined based on the Nyquist impedance diagrams, Z″(Z′) and of the fit parameters corresponding to the equivalent electrical model for each sample and the results show that by increasing the volume fraction x from 0 to 5.1%, the σ_DC_ increase from 2.637·10^−8^ S/m to 5.725·10^−8^ S/m.

Using the obtained values σ_DC_ and the Mott’s variable-range-hopping (VRH) model, we determined the hopping distance R_h_ of the charge carriers within the samples, which decreases with the increasing of volume fraction from 7.30 nm (for x = 0) to 5.92 nm (for x = 5.1%). At the same time, we evaluated the hopping frequency (ω*_h_*), from the DC regime (at low frequencies) to the dispersive regime (at high frequencies), which increases from 7.625·10^3^ s^−1^ (for x = 0) to 10.890·10^3^ s^−1^ (for x = 5.1%).

The frequency dependencies of dielectric permittivity show that the imaginary component ${\varepsilon^{\prime \prime }}_{rel}$ has a maximum at frequencies close to the frequencies of the maximum of M″ which indicates the existence of an interfacial relaxation process.

This study shows that the electrical conduction process and the dielectric properties of silicone rubber/Al particles’ composite samples can be significantly changed by the volume fraction of Al particles, being useful in the design of flexible electronic applications.

In our future research, we will expand the study of the electrical and dielectric properties of these composites, by using other types of metal particles or silicone rubber, following the dependence both of the volume fraction and the temperature, of the σ and of ε, based on the complex impedance measurements in different frequency and temperature ranges.

## Figures and Tables

**Table 1:** Electrical parameters of composite samples, determined from measurements.

Samples	A (x = 0)	B (x = 0.5%)	C (x = 1%)	D (x = 2.5%)	E (x = 5.1%)
$\tau_{Z}\;[ms]$	0.925	0.763	0.558	0.413	0.330
*R*[Ω]	1.5166∙10^8^	1.3203∙10^8^	1.0841∙10^8^	8.2876∙10^7^	6.987∙10^7^
*P*[s^k^/Ω]	6.7444∙10^−12^	7.1842∙10^−12^	7.3427·10^−12^	7.5766∙10^−12^	7.6777∙10^−12^
*k*	0.95118	0.94992	0.94841	0.94638	0.94593
$\tau_{M}\;[ms]$	0.496	0.447	0.370	0.292	0.237
$\tau_{rel}\;[ms]$	0.529	0.476	0.456	0.446	0.413
$\sigma_{DC}\;[S/m]$	2.637·10^−8^	3.029·10^−8^	3.689·10^−8^	4.826·10^−8^	5.725·10^−8^
*n*	0.593	0.579	0.561	0.534	0.515
*A_0_* [S/ms^n^]	1.315·10^−10^	1.687·10^−10^	2.282·10^−10^	3.566·10^−10^	4.772·10^−10^
$\omega_{c}[s^{-1}]$	7.625·10^3^	7.820·10^3^	8.647·10^3^	9.803·10^3^	10.890·10^3^
$R_{h}\;[nm]$	7.30	6.89	6.57	6.11	5.92

**Table 2:** The maximum relative change of the capacitance.

f [Hz]	$\left(C_{x\max}-C_{x0})/C_{x0}\;[\%\right]$
100	24.3
200	16.9
500	13.0

## Data Availability

The data presented in this study are available upon request from the corresponding author.
